# Supplementary material for: Fluid migrations and volcanic earthquakes from depolarized ambient noise
Source: Nat Commun. 2021 Nov 17;12:6656. doi: 10.1038/s41467-021-26954-w (PMC8599670; doi:10.1038/s41467-021-26954-w)
Supplement: Supplementary file 1 — Supplementary Information [file 41467_2021_26954_MOESM1_ESM.pdf]

Supplementary Information for:

Fluid migrations and volcanic earthquakes from depolarized ambient noise

Petrosino, S.[1] and De Siena, L.[2,3,*]

[1] *Istituto Nazionale di Geofisica e Vulcanologia, Sezione di Napoli - Osservatorio Vesuviano, Napoli, 80124, Italy.*

[2] *Institute of Geosciences, Johannes Gutenberg University, Mainz, 55128, Germany.*

[3] *Terrestrial Magmatic Systems Research Consortium, Johannes Gutenberg University, Mainz, 55128, Germany.*

* *ldesiena@uni-mainz.de*

Supplementary Figures

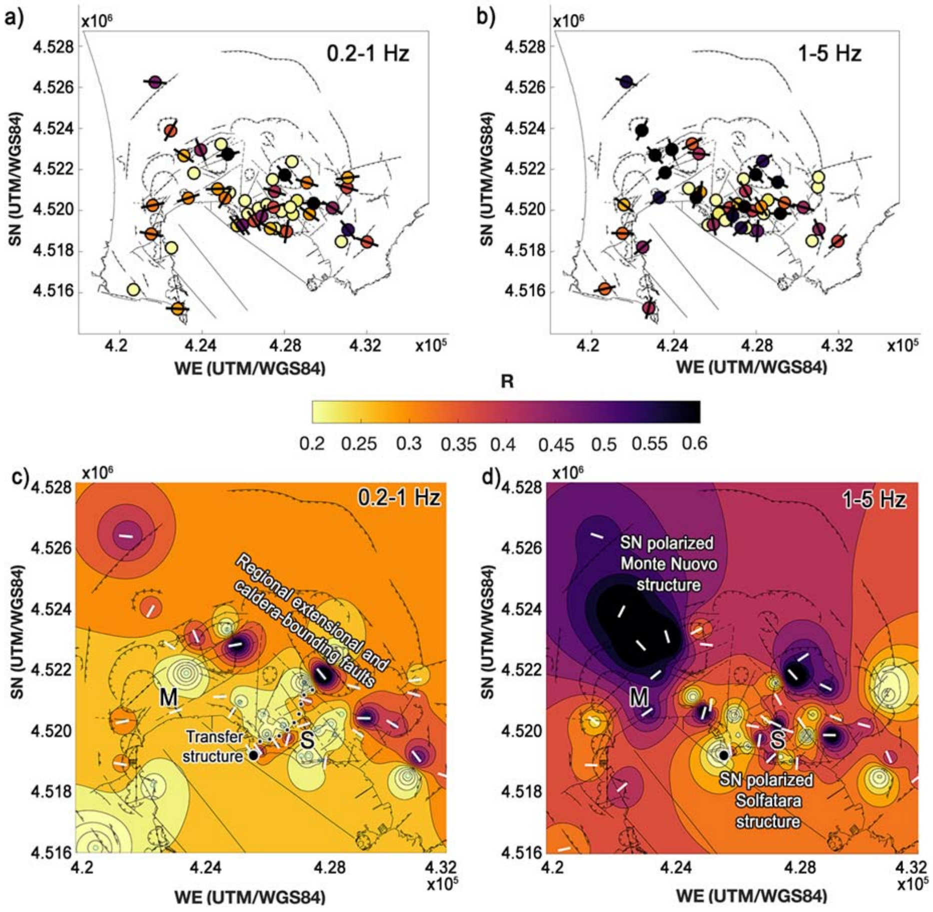

Supplementary Figure 1. Resultant lengths, azimuths, and interpolated mapping Resultant lengths (*R*, coloured circles) and azimuths (only for *R*>0.25) obtained at Campi Flegrei in periods of low seismic release (2009 and 2017) in the 0.2-1 Hz (a) and 1-5 Hz (b) frequency bands. The patterns are imposed over fault strikes, fractures, and craters. c-d) The resultant length has been plotted with a squared interpolation from each station. Azimuths are over imposed as white lines. The black dot is the stationary point of maximum vertical deformation in the last 36 years. The Solfatara crater (S) and Monte Nuovo (M) are marked on the maps.

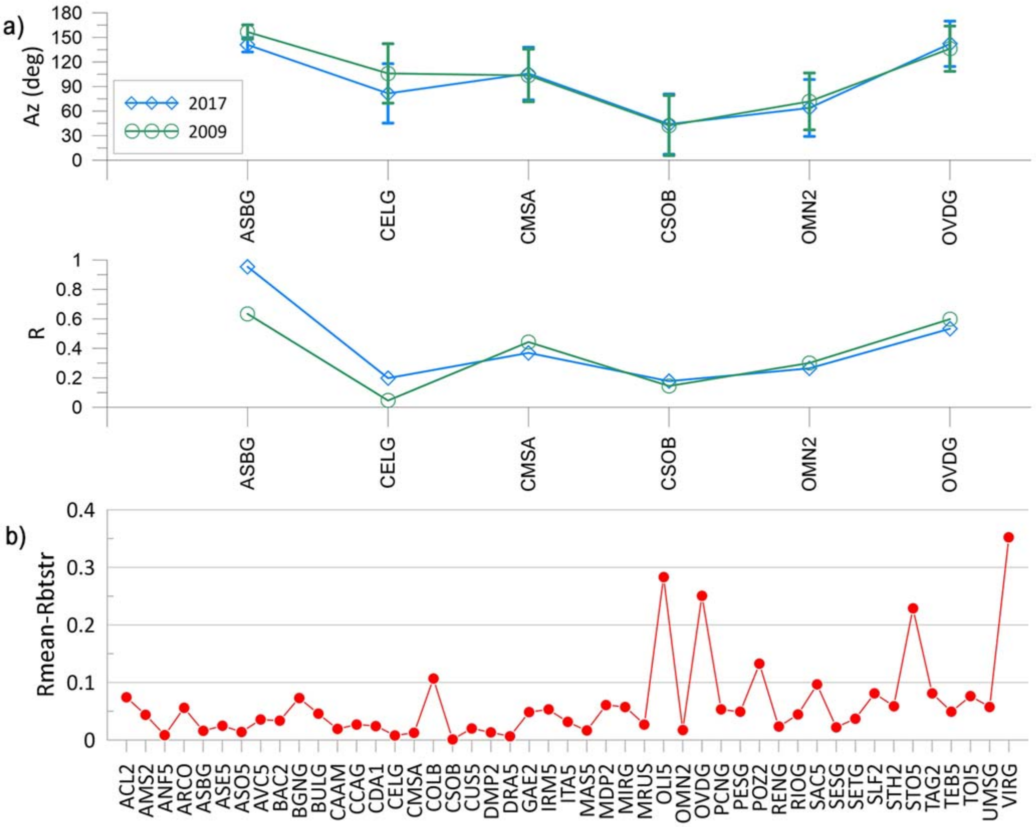

Supplementary Figure 2. Stability of the results obtained between 2009 and 2017. a) Azimuth and *R* at stations recording in the two periods. Error bars are equal to one standard deviation. b) A bootstrap test applied to *R* estimates for the 47 stations recording across the two periods.

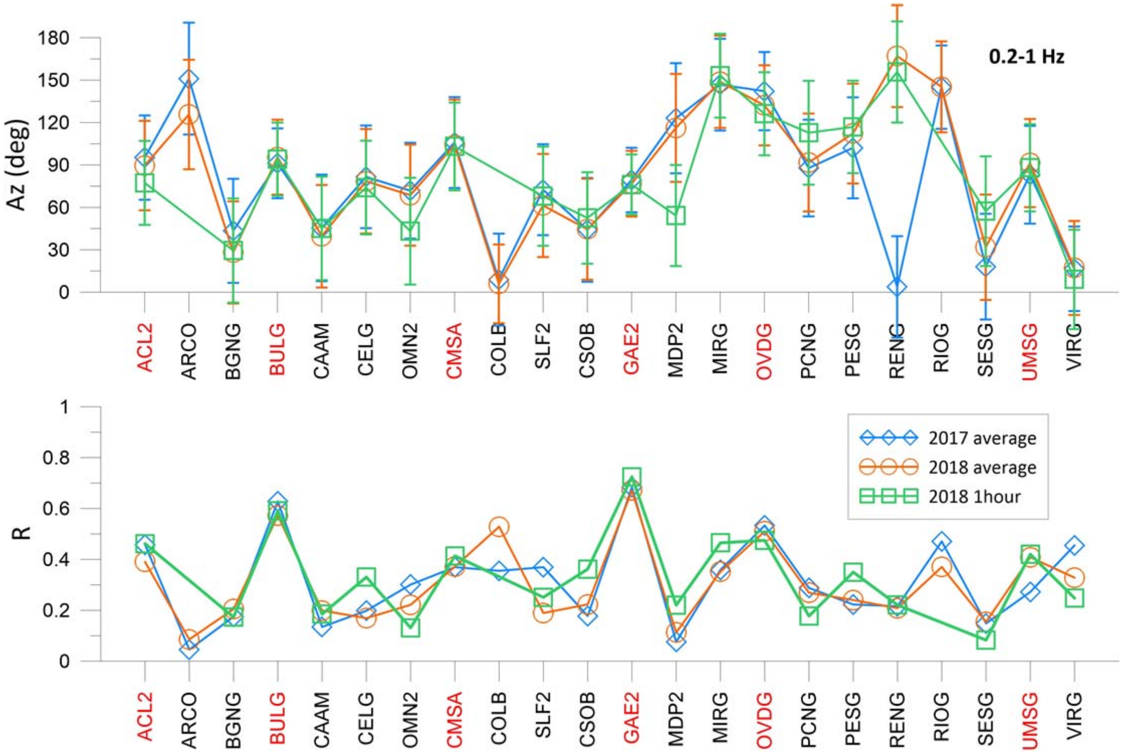

Supplementary Figure 3. Comparison between resultant lengths and azimuths: The stability of *R* and azimuths is evaluated computing these parameters over six months (2017 and 2018) and one hour (2018) at co-located stations. Stations on the extensional trend are labelled in red. Error bars are equal to one standard deviation.

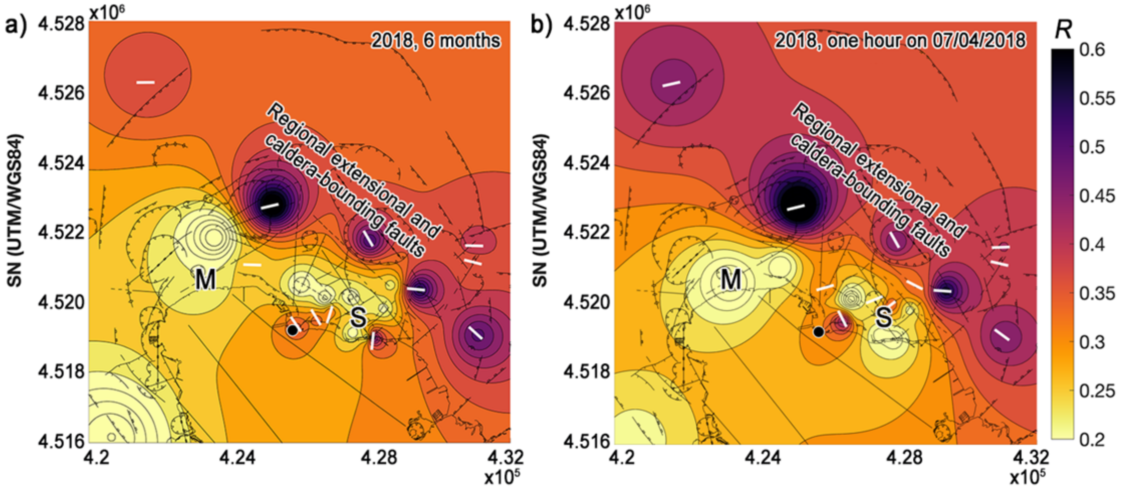

Supplementary Figure 4. Resultant lengths in 2018. The stability of *R* and azimuths is evaluated computing these parameters over a) six months and b) one hour in 2018 at co-located stations.

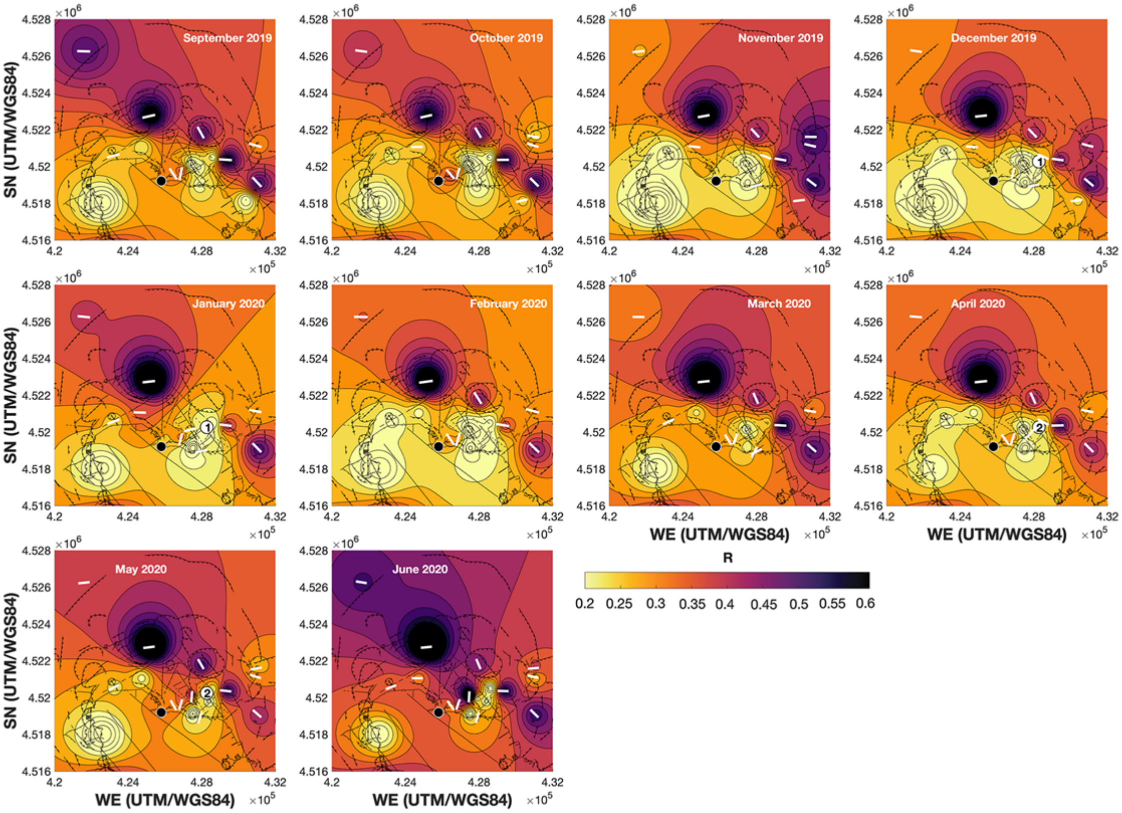

Supplementary Figure 5. Monthly variations for the pre-, inter-, and post-seismic period. The figure shows monthly maps of R between September 2019 and June 2020. Each panel shows the interpolated measurements taken over one month.

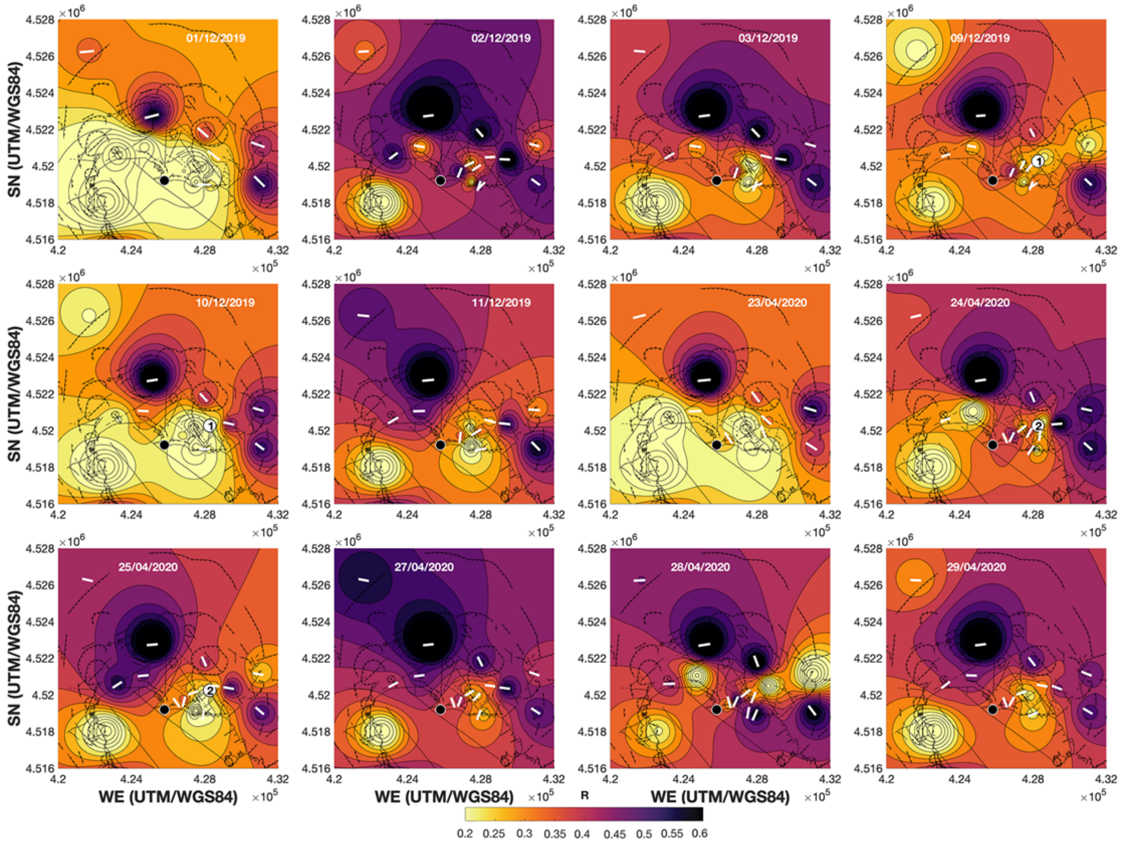

Supplementary Figure 6. Daily variations for the pre-, inter-, and post-seismic period. Each panel shows daily interpolated measurements of *R* taken across December 2019 and April 2020.

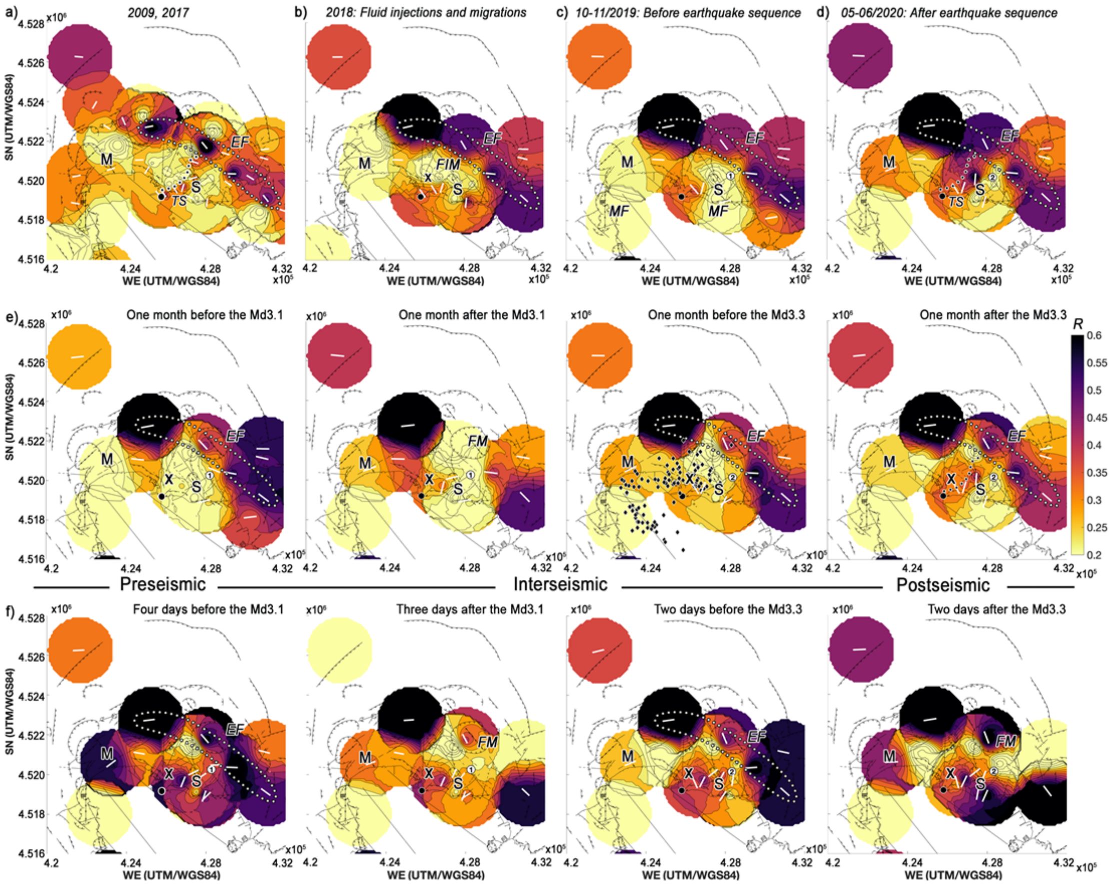

Supplementary Figure 7. Results of the polarization analysis with a different interpolation. Resultant lengths (*R*, coloured circles) and azimuths (only for *R*>0.25) are imposed over fault strikes, fractures, and craters. The polarization maps are obtained with a squared interpolation of ray equal to one wavelength (1.7 km), for an average shear-wave velocity of 1.2 km/s[16] and dominant frequency of 0.7 Hz. a -d) Equivalent to Fig. 1a-d. e-f) Equivalent to Fig. 3a,b.

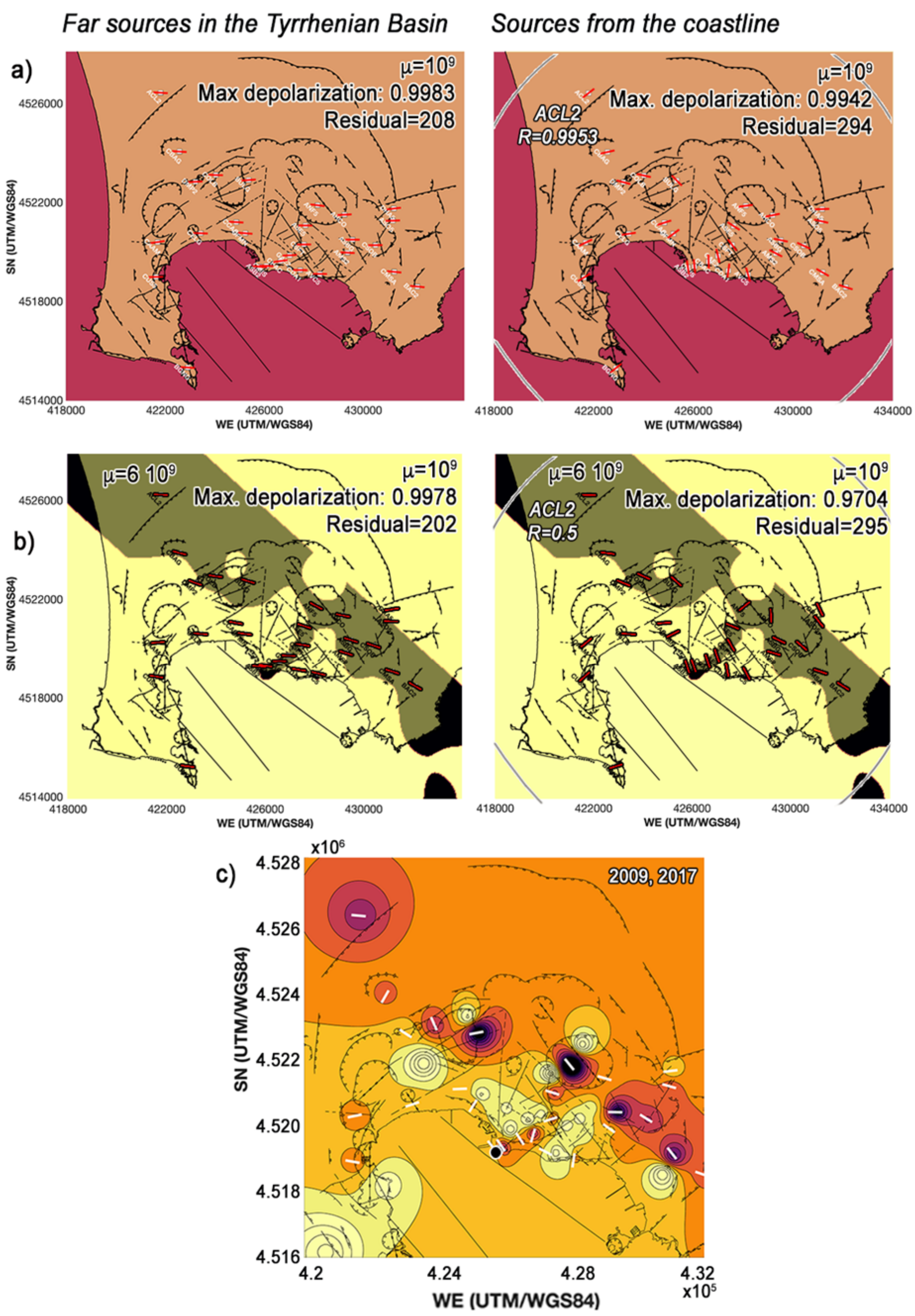

Supplementary Figure 8. Changes in polarization between a homogeneous medium and an increase in shear modulus led by the results for two source settings. a) Shear modulus, resultant lengths and azimuths corresponding to sources far in the Tyrrhenian Basin. The left panel has constant shear modulus. Polarization parameters are calculated over 70 seconds. The right panel shows the results with an increase of shear modulus (50%) assigned to the propagation grid based on resultant lengths (R>=0.31) and increase in velocity from ambient noise tomography. b) Same as panel a) for circular sources giving a long-wavelength representation of sources at the coastline. Only in this case the azimuths become perpendicular to the transfer connection. c) The results of the two simulations ae compared with the analysis in 2009 and 2017.

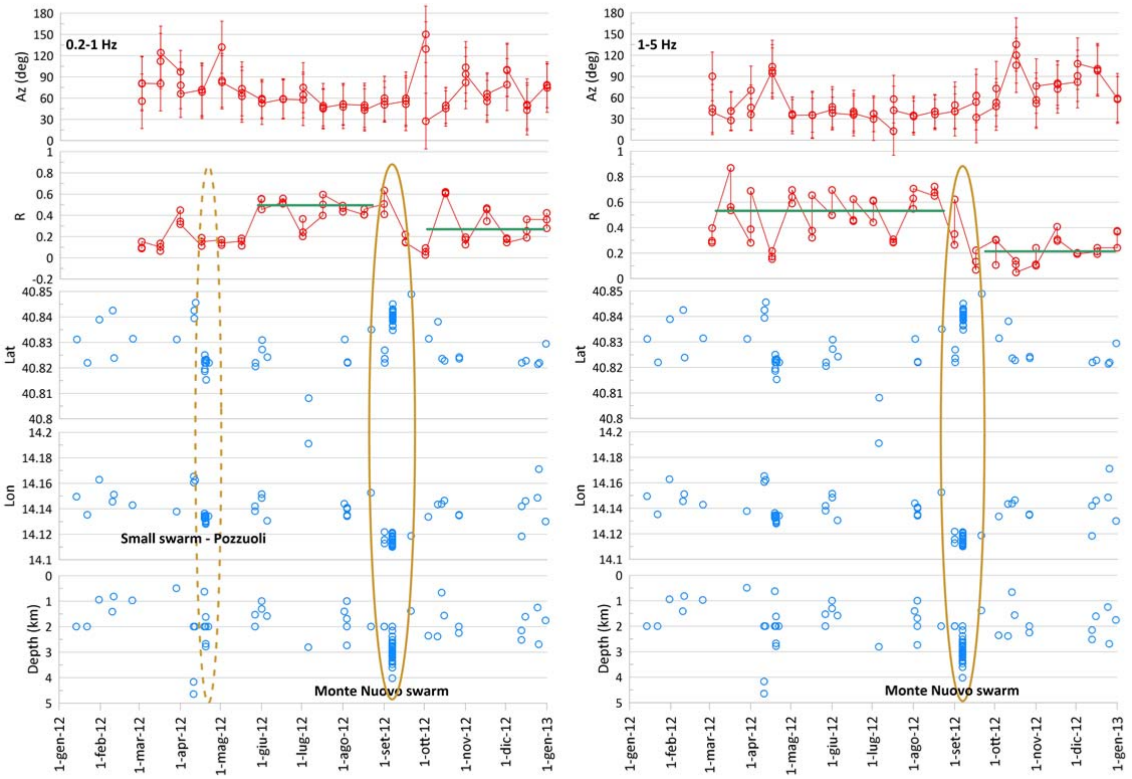

Supplementary Figure 9. Permanent reduction of the polarization parameters after seismic swarms. The graphs compare the variations of the polarization parameters with the locations and depths of swarms at Campi Flegrei in 2012. The Monte Nuovo swarm of September 2012 (Fig. 2b, western seismicity) and the corresponding decrease of the resultant length (*R*) are marked by an ellipse (continuous line) on the graphs. In the 0.2-1 Hz frequency band, the small Pozzuoli swarm of April 2012 also produces a variation of *R* (left panel, dotted ellipse). Trend lines before and after the Monte Nuovo swarm are drawn in green. Error bars are equal to one standard deviation.

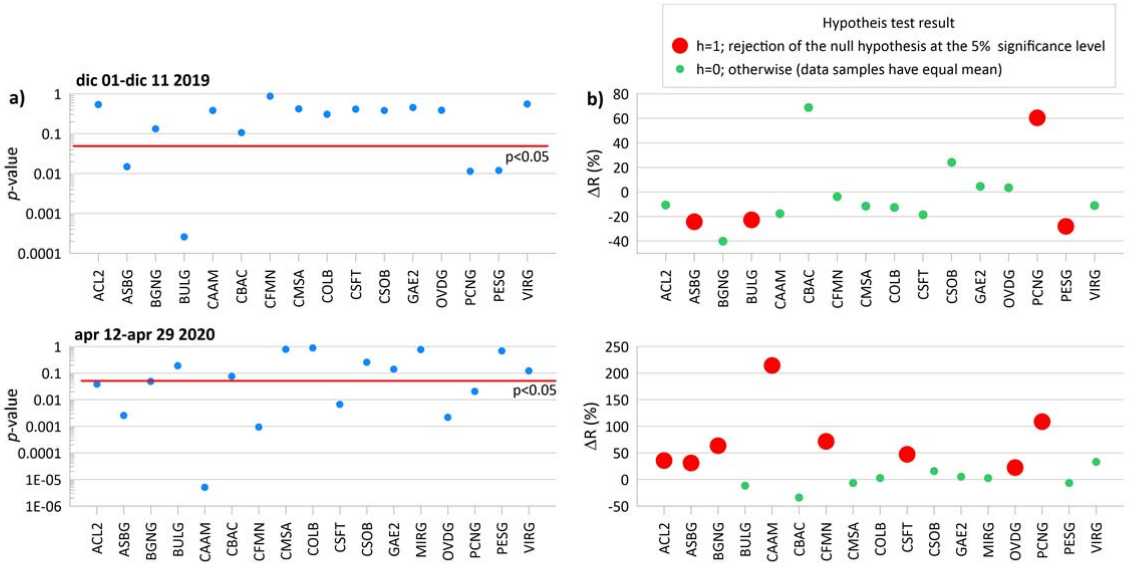

Supplementary Figure 10. Statistical significance of polarization variations after earthquakes. The graphs show the results of the t-test at the same stations before and after the Md3.1 (December 6th, 2019) and the Md3.3 (April 26th, 2020) earthquakes in the 0.2-1 Hz frequency band. a) p-values <0.05 confirm the hypothesis that the two sample populations (before and after the earthquakes) have different means. b) Percentage variation of the resultant length (*R*) before and after the earthquakes; red full circles mark the statistically significant changes.

Supplementary Table

Supplementary Table 1: Physical parameters of the numerical simulations.

Notation	*Value*	*Description*
f	0.7 Hz	Dominant Frequency
Δl	40 m	Grid step
τ	100 s	Max. record time
Δt	1 ms	Time step
(WE,SN)	(16,16) km	Solution grid dimensions
(LWE_{abs}, LSN_{abs})	(14,14) km	Length of absorbing boundaries.
μ^{h}	1 GPa	Homogeneous shear modulus[17]
μ^{i}	6 GPa	Increased shear modulus[17]
Q	30	Homogeneous Q factors[25]
ρ	2500 kg/m^3	Density[4]
λ^{h}	0.9 km	Homogeneous wavelength
λ^{i}	2.2 km	Increased wavelength
